# Toward enhanced understanding and rigor: addressing limitations in studying dementia symptoms and caregiver interventions

**DOI:** 10.1590/1980-5764-DN-2024-0148

**Published:** 2024-11-29

**Authors:** Guilherme Nobre Nogueira, Wilson da Silva Rocha Vidal, Nicole Custódio Porto Silva

**Affiliations:** 1 Universidade Federal do Ceará, Faculdade de Medicina, Departamento de Medicina Clínica, Fortaleza CE, Brazil; 2 Universidade Nove de Julho, Faculdade de Medicina, Departamento de Medicina Clínica, São Paulo SP, Brazil; 3 Universidade Federal da Paraíba, Faculdade de Medicina, Departamento de Medicina Clínica, João Pessoa PB, Brazil

Dear Editor,

The article entitled “Behavioral and Psychological Symptoms of Dementia in Long-Term Care Setting: Assessment of Elderly Adults and Intervention for Caregivers,” by Duarte et al.^1^, published in Dementia and Neuropsychologia on November 27, 2023, significantly contributes to the literature on the psychological symptoms of dementia in long-term care settings. However, given the future rise in the prevalence of dementia in the growing elderly population, certain limitations, methodological challenges, and inquiries regarding the qualitative approach of such studies deserve attention to provide more robust evidence regarding the effectiveness of these interventions.

Firstly, no distinction is made for confounding factors for the parameters analyzed in the article, such as mentioning the genders of dementia patients. In this regard, it would be important for the authors to adequately report confounding variables that may influence results, such as participants’ demographic characteristics, comorbidities, and other factors that may interfere with the outcomes^2,3^.

Secondly, considering that the addressed patients belong to diverse cultural and social contexts, the study fails to implement a randomized controlled trial design, where participants are randomly assigned to an intervention group and a control group. This would help minimize selection bias and establish stronger causal relationships^4,5^.

Thirdly, to better assess intervention efficacy, it would be beneficial to conduct long-term follow-ups to determine whether observed positive effects are sustained over time or if there are long-term changes in caregivers’ and patients’ well-being^6,7^.

Fourthly, there is no standardization in the psychoeducational intervention, including content, duration, frequency, and methodology. A rigorous standardization of the intervention would help ensure consistency in delivery and implementation.

In conclusion, acknowledging the valuable contribution of the study amid an alarming future, addressing the aforementioned limitations, will strengthen its internal and external validity, reliability, and relevance of the obtained results. This will enhance the study’s rigor and facilitate a more nuanced understanding of the implications of psychological symptoms of dementia in long-term care settings within the scientific community, enabling a more widespread implementation of caregiver qualification and training interventions for dementia patients.
